# Actual Incidence and Clinical Behaviour of Follicular Thyroid Carcinoma: An Institutional Experience

**DOI:** 10.1155/2014/952095

**Published:** 2014-03-04

**Authors:** Carmela De Crea, Marco Raffaelli, Luca Sessa, Simona Ronti, Guido Fadda, Chiara Bellantone, Celestino Pio Lombardi

**Affiliations:** ^1^Istituto di Semeiotica Chirurgica, U.O. di Chirurgia Endocrina e Metabolica, Università Cattolica del Sacro Cuore, Policlinico A. Gemelli, L.go A. Gemelli 8, 00168 Rome, Italy; ^2^Istituto di Anatomia Patologica, U.O. di Istologia e Citodiagnosi, Università Cattolica del Sacro Cuore, Policlinico A. Gemelli, L.go A. Gemelli 8, 00168 Rome, Italy

## Abstract

Follicular thyroid carcinoma classically accounts for 10–32% of thyroid malignancies. We determined the incidence and the behaviour of follicular thyroid carcinoma in an endemic goitre area. A comparative analysis between minimally invasive and widely invasive follicular thyroid carcinoma was performed. The medical records of all patients who underwent thyroidectomy from October 1998 to April 2012 for thyroid malignancies were reviewed. Those who had a histological diagnosis of follicular carcinoma were included. Among 5203 patients, 130 (2.5%) were included. Distant metastases at presentation were observed in four patients. Sixty-six patients had a minimally invasive follicular carcinoma and 64 a widely invasive follicular carcinoma. In 63 patients an oxyphilic variant was registered. Minimally/widely invasive ratio was 41/26 for *usual follicular carcinoma* and 25/38 for oxyphilic variant (*P* < 0.05). Patients with widely invasive tumors had larger tumors (*P* < 0.001) and more frequently oxyphilic variant (*P* < 0.05) than those with minimally invasive tumours. No significant difference was found between widely invasive and minimally invasive tumors and between *usual follicular carcinoma* and oxyphilic variant regarding the recurrence rate (*P* = NS). The incidence of follicular thyroid carcinoma is much lower than classically retained. Aggressive treatment, including *total thyroidectomy* and radioiodine ablation, should be proposed to all patients.

## 1. Introduction

Follicular thyroid carcinoma (FTC) is the second most common thyroid malignancy after papillary thyroid carcinoma (PTC) [1, 2]. Classically, the reported incidence of FTC is highly variable ranging from 10% to 32% of *differentiated thyroid carcinoma* [2–11]. However, there are marked geographical variations in the relative proportions of FTC, likely due to difference in dietary iodine content. Indeed, in iodine-deficient areas the relative rate of FTC tends to be even higher, up to 40% of the cases of *differentiated thyroid carcinoma* [1, 2, 8, 12, 13].

The wide range of reported incidence could be, at least in part, explained by an interobserver variability in the histopathological diagnosis of FTC [14–16].

However, more recently, a decreased incidence of FTC has been reported [14, 17]. This decrease is probably due to a more accurate histological diagnosis (e.g., exclusion of atypical follicular adenoma, identification of follicular variants of PTC) and also to iodine supplementation programs [2, 14, 17].

Traditionally, FTC has been classified as minimally invasive (MI-FTC) and widely invasive (WI-FTC) [17–19].

The World Health Organization (WHO) stated that MI-FTC has limited capsular and/or vascular invasion whereas WI-FTC has widespread infiltration of adjacent thyroid tissue and/or blood vessels [17]. Although this classification is generally well accepted, definitions of the extent of capsular invasion may be variable in different settings [2, 15]. This lack of uniformity can make it difficult to compare different patient series and this may imply clinical uncertainty regarding prognosis [18, 20–22]. Moreover, the influence of vascular invasion on FTC prognosis remains controversial, since some authors have proposed a further classification of FTC adding the group of moderately invasive FTC, with an intermediate prognosis between MI-FTC without vascular invasion and WI-FTC [2].

The clinical implications of these subclassifications would be relevant as they potentially could involve different treatment options [15, 23].

Indeed, some studies have reported a very small risk of recurrent disease or distant metastasis in patients with MI-FTC and therefore a limited thyroid resection (e.g., thyroid lobectomy) has been suggested as adequate treatment in such cases [15, 16, 21, 24]. On the contrary, other studies demonstrating the occurrence of distant metastasis also in MI-FTC indicated *total thyroidectomy* and radioiodine ablation as the treatment of choice for all the patients with FTC [3, 8, 15, 25, 26]. Further, age and size have been evaluated as prognostic factors, possibly influencing the extension of the surgical resection [15, 27, 28].

In addition, some evidence in the literature has focused on the prognostic implications and the treatment outcomes of the Hürthle cell carcinoma (HCC) [29–32]. Although HCC has been classified by the WHO as a variant of FTC [17] and it is treated as such according to the American Thyroid Association (ATA) management guidelines [33], some authors believe that it is a different and more aggressive tumour [32, 34]. As a consequence, the optimal management of patients with this tumour has been issue of considerable controversy [29].

The uncertainty surrounding the best management practice and the controversy in the prognostic significance of the histopathological characteristics make it difficult for clinicians to determine the most suitable treatment recommendations for FTC.

The aim of this study was to review the experience with FTC in a tertiary referral centre and to determine the actual incidence and the clinical behaviour of FTC in an endemic goitre area.

## 2. Materials and Methods

### 2.1. Patients Population

All patients who underwent surgery for *differentiated thyroid carcinoma* in our institution were prospectively registered in a specifically designed database (Microsoft Excel, Microsoft Corporation, Redmond, WA, USA). Among all the patients registered, those with diagnosis of FTC who underwent thyroidectomy between October 1998 and April 2012 were reviewed for the purpose of the present study. Histological specimens of the selected patients were reexamined by an experienced pathologist (Guido Fadda) using the WHO classification [17].

The following parameters were analyzed for the purpose of the present study: age, sex, surgical procedure, pathological lesion size, pathological diagnosis, TNM staging [35], and follow-up evaluation results.

### 2.2. Study End Points

The primary end point was to analyze the clinical outcomes of patients with FTC treated at our institution. Secondary end points of the study were the determination of the actual incidence of FTC in an endemic goitre area and the comparative evaluation of different subtypes of FTC (MI-FTC versus WI-FTC and HCC versus non-HCC).

### 2.3. Definitions

The histological diagnosis and classification of FTC in this study were based on the most recent WHO classification [17]. The presence of capsular and/or vascular invasion and the absence of nuclear features of PTC defined pathological diagnosis of FTC. Follicular variants and oxyphilic cell variant of PTC were specifically excluded. Moreover, cases diagnosed as poorly differentiated carcinoma on the basis of WHO classification [17] and those including anaplastic component were excluded from this series. When limited capsular and/or vascular invasion were observed, FTC was classified as MI-FTC, whereas when wide infiltration of adjacent thyroid tissue and/or blood vessels was observed, FTC was classified as WI-FTC. FTC was classified as HCC when more than 75% of the cells were Hürthle cells and there was invasion through the entire thickness of the tumour capsule and/or invasion of blood vessels within the capsule or adjacent to it [17].

Loboisthmusectomy implies the complete extracapsular resection of a thyroid lobe with more than one-half of the isthmus (including the pyramidal lobe). *Total thyroidectomy* is the total bilateral extracapsular lobectomy. Completion thyroidectomy is the resection of all residual thyroid tissues after a previous partial resection. *Central compartment node dissection* implies complete level VI neck node dissection. *Lateral neck node dissection* implies levels II to V neck node dissection.

All the surgical procedures were performed by an experienced endocrine surgeon or by a resident operating under supervision.

Pathological tumour staging was defined in accordance with the 2010 7th edition of the American Joint Committee on Cancer pTNM staging system [35].

Distant metastasis (disease outside the thyroid bed and cervical lymph node) was identified by biopsy of extrathyroidal sites demonstrating FTC or in the presence of an elevated serum thyroglobulin (sTg) and/or sites of uptake on postoperative radioactive iodine scans, eventually confirmed by the means of radiological studies.

### 2.4. Management Strategy and Follow-Up Evaluation

The management of patients with FTC included primary surgery followed by an evaluation for ^131^I ablation (RAI) on the basis of stage and risk factors, according to the ATA [33].

The protocol for the postoperative management and follow-up evaluation of patients with *differentiated thyroid carcinoma* has been previously described in detail [36]. Briefly, postoperative suppressive levothyroxine (LT4) treatment was administered to all the cases. All the patients underwent sTg and anti-Tg antibody measurements under suppressive LT4 treatment and an ultrasound (US) neck scan 3 to 6 months after surgery. This was the only follow-up protocol adopted for patients with pT1 *differentiated thyroid carcinoma *≤1.0 cm, in the absence of lymph node metastases and multifocality.

Low-risk patients were evaluated by ^131^I diagnostic whole body scans and TSH-stimulated sTg. All of the high-risk patients underwent RAI. Patients who did not receive RAI underwent long-term follow-up at 6–12-month intervals, at which time an US scan was performed and sTg levels were measured while the patients were on LT4.

Follow-up data were obtained by outpatient consultations or telephone contact. For the evaluation of the oncologic outcome only patients with completed follow-up were considered.

### 2.5. Statistical Analysis

Statistical analysis was performed using a commercially available software package (SPSS 15.0 for Windows, SPSS Inc., Chicago, IL, USA). The *χ* ^2^ test was used for categorical variables, and the *t*-test was used for continuous variables. A *P* value less than 0.05 was considered significant.

A comparative analysis between MI-FTC and WI-FTC and between HCC and non-HCC tumours concerning the registered parameters was performed. Risk factors for distant metastasis were evaluated by univariate analysis.

## 3. Results

During the study period, 5203 patients underwent surgery for *differentiated thyroid carcinoma*. Among these, 130 (2.5%) had a histopathological diagnosis of FTC, confirmed at rereview pathological examination. There were 39 males and 91 females with a mean age of 51.1 ± 14.5 years (range: 18–88) (Table 1).

The characteristics of all the included patients are reported in Table 1.

All the patients underwent *total thyroidectomy.* In nine patients (6.9%) a two-stage *total thyroidectomy* (lobectomy plus subsequent completion thyroidectomy) was performed because of the diagnosis of FTC at the initial histological examination: pT2 FTC in 8 cases and pT3 FTC in 1 case, with concomitant PTC in 2 out of nine cases (unilateral multifocal PTC and micro-PTC, resp.).

One patient (0.8%) with a preoperative diagnosis of lateral neck node metastases underwent *central compartment node dissection* and unilateral *lateral neck node dissection.*

According to WHO classification [18], 66 (50.8%) patients had a MI-FTC and 64 (49.2%) patients a WI-FTC.

HCC was observed in 63 cases (48.5%) (Table 1).

The mean pathological lesion size was 28.9 ± 16.6 mm (range: 5–100). Final histology revealed 36 (27.7%) pT1 (7 microcarcinomas, 2 multifocal diseases), 63 (48.5%) pT2, and 31 (23.8%) pT3 (2 multifocal diseases).

Four patients (3.1%) had distant metastases at presentation: bone metastasis in two cases, chest wall metastasis in one case, and lung metastasis in the remaining case.

Follow-up evaluation was completed in 117 out of 130 patients (90%). The mean follow-up period was 103.9 ± 39.5 months (range: 17–182).

Overall 105 (89.7%) patients underwent RAI under hypothyroid condition (TSH > 30 *μ*UI/mL). In the remaining 12 patients, follow-up evaluation only included basal sTg measurements and neck US scan: mean sTg level under LT4 suppressive treatment was undetectable in all the patients and the US evaluation showed no thyroid remnants or lymph node involvement in all these cases.

No cancer-related deaths were registered. Four patients (3.4%) *died from unrelated causes.*

Distant metastases were registered in three cases (2.6%): one patient with bone metastases at presentation had lung and kidney metastases 16 months after the initial diagnosis; two patients developed, respectively, lung and bone metastases, 4 months and 36 months after surgery.

No significant difference was found between MI-FTC and WI-FTC patients concerning age, sex, N stage, M stage, multifocal disease, and association with PTC (*P* = NS).

The mean tumour size was significantly larger in WI-FTC than in MI-FTC (35.1 ± 17.8 versus 23.1 ± 13.0 mm) (*P* < 0.001) (Table 1). No statistically significant difference between MI-FTC and WI-FTC was found regarding the recurrence rate (*P* = NS) (Table 1).

No significant difference was found between HCC and non-HCC patients concerning sex, T stage, N stage, and multifocal disease (*P* = NS) (Table 2). The mean age of HCC patients was significantly higher than that of non-HCC (55.0 ± 14.4 versus 47.5 ± 13.8 years) (*P* < 0.01). The rate of distant metastasis at presentation was 1.6% (1/63) and 4.4% (3/67), respectively, in HCC and non-HCC patients (*P* = NS). The mean tumour size of HCC was significantly larger than that of non-HCC patients (32.4 ± 18.9 versus 25.6 ± 13.5 mm) (*P* < 0.05). The WI pattern was significantly more frequent in HCC than in non-HCC tumours (38/63 versus 26/67) (*P* < 0.05). *All the patients in whom recurrent disease was observed had HCC tumour *(*P* = NS) (Table 2).

None of the analyzed parameters significantly differed between patients with and without distant metastases at univariate analysis (Table 3).

## 4. Discussion

FTC and PTC are often analyzed together in most of the reports because of the similarities in their clinically indolent behaviour, management, and outcome. In clinical practice, however, the presentation of PTC and FTC differs markedly [3, 8, 10]. Indeed, patients with FTC generally are older, present with more advanced disease, and have a poorer prognosis [3, 8, 10, 11]. More recent studies tend to stratify patient outcome according to histologic classification. In the present study we analyzed the clinical features and outcomes of patients with FTC who were managed at a single institution in an endemic goitre region. The present series is characterized by a considerable homogeneity in treatment and histopathological diagnosis, since all the patients were operated on in the same institution and the histological specimens were reexamined by an experienced pathologist using the WHO classification [17].

Since an increased risk of FTC has been reported in areas of iodine deficiency and in areas of endemic goitre, iodine deficiency and endemic goitre are thought to be predisposing factors to the development of FTC [37]. *Based on urinary iodine excretion, the International Council for Control of Iodine Deficiency Disorders classified Italy as a country with deficient iodine nutrition, since the median urinary iodine excretion was *<100 *μ*g/L [38]. Classically, the reported incidence of FTC is up to 40% in iodine-deficient region [8, 12, 13]. Among 5203 consecutive *differentiated thyroid carcinomas* that were managed in our institution over 14 years, the rate of FTC was 2.5%. This rate is markedly lower than classically reported [8, 12, 13]. However, such findings are in agreement with recent studies that reported a significant decrease in FTC incidence [39–41]. Several explanations may be offered for this occurrence. First, a shift in the PTC/FTC ratio is a well-recognized effect of iodine prophylaxis, which has been observed also in Italy in recent years [33, 39]. Another explanation of the decreased incidence of FTC could be the greater attention to the histological pattern of the follicular variant of PTC [14–16, 39]. Albores-Saavedra et al. [42] reported that the rate of this variant increased by 173% between 1973 and 2003, suggesting that this could be the major contributing factor to the decrease of FTC in the last decades.

In the present series, according to the WHO definition [17], FTCs were classified in MI-FTC and WI-FTC based on the degree of disposition to invade the surrounding tissues and vessels.

On the basis of this classification, a quite similar rate of MI-FTC and WI-FTC (50.8% versus 49.2%, resp.) was observed in this series. Similar to other experiences [15], we hypothesized that this could be explained by the strict criteria used for the classification of MI-FTC and WI-FTC and the exclusion of tumours with insular or anaplastic characteristics.

On the other hand, contrary to other findings suggesting that MI-FTC occurs more frequently in younger patients [8, 21, 43], in the present series, we found no significant difference in terms of age between patients with MI-FTC and with WI-FTC.

Lymph node involvement in FTC is uncommon (0–10%) [15, 21]. This was confirmed also by the present series. Indeed, only one patient out of 130 included (0.7%) underwent therapeutic *central compartment node dissection* and unilateral *lateral neck node dissection*, for preoperatively demonstrated lateral neck nodes metastases. Among all the other patients no one underwent any neck dissection. At a mean follow-up of about 9 years none of them developed neck nodal recurrence.

On the basis of these results we would continue to suggest that nodal dissection in FTC should be performed only in case of suspicion of node metastases, with therapeutic intent.

In the case oxyphilic cells represent more than 75% of an encapsulated nodule, the lesion is classified as a Hürthle cell neoplasm. HCC has generally been considered to be a variant of FTC [17, 30]. Our study, as well as others, has shown that HCC occurs in an older age group than non-HCC does [30]. Several studies have reported that HCC is more aggressive and has a poorer prognosis than non-HCC [29, 30]. In our experience, distant metastasis diagnosed during follow-up developed in all the cases in HCC patients, even if these results did not reach the statistical significance.

In the present series, distant metastases, either at presentation or as recurrent disease, were observed in six patients. In agreement with other reports [43], large tumour size was not a risk factor for distant metastases. Indeed, tumour size was not significantly different between patients with and those without distant metastases. Moreover, in the present series among 6 patients with distant metastases, 2 (33.3%) had tumours ≤2.0 cm in their maximum diameter (pT1 FTCs).

On the other hand, no association between distant metastasis and pattern of growth (MI versus WI) was observed in our experience. This finding is in disagreement with previous reports [2, 3, 8]. However, it could be related to the fact that the number of patients who presented distant metastases is low. Indeed, no patients with MI-FTC developed distant metastases during the follow-up, even if one of them had distant metastases at diagnosis. Conversely, among patients with WI-FTC, 3 had metastases at presentation and 3 developed distant metastases during follow-up. Recurrent distant metastatic disease was diagnosed by radioiodine scan in all the patients. This finding led us to propose, in agreement with other authors [3, 8, 25], *total thyroidectomy* and radioiodine remnant ablation in all the patients with FTC in order to facilitate the diagnosis and management of distant metastatic disease.

Indeed, although several studies have suggested that patients with MI-FTC and no vascular invasion might benefit from a more conservative treatment (e.g., thyroid lobectomy) [2, 15, 16, 21, 24], we retain that a more aggressive approach (i.e., *total thyroidectomy* in all the patients) could be suitable. This approach has the advantage to facilitate follow-up and the detection of occult distant metastases that could occur at presentation even in patients with MI-FTC without vascular invasion [3, 8, 15, 25, 26].

## 5. Conclusions

The incidence of FTC is much lower than reported, even in a region of iodine deficiency and with endemic goitre.

No clinical and pathological prognostic factors were able in our experience to individualize treatment, identifying patients that might be safely treated with less than *total thyroidectomy.* Aggressive treatment, including *total thyroidectomy* and RAI, should be proposed to all the patients. Prophylactic neck node dissection should not be recommended.

## Figures and Tables

**Table 1:** Clinical, pathological, and follow-up data of all the included patients. Comparative analysis between MI-FTC and WI-FTC.

	All patients	MI-FTC	WI-FTC	*P* value
Patients	130	66	64	
Age (±SD^a^) (range) years	51.1 ± 14.5 (18–88)	50.0 ± 11.6 (28–76)	52.3 ± 9.9 (18–88)	NS*
Male/female	39/91	15/51	24/40	NS*
Tumour size (± SD^a^) (range) mm	28.9 ± 16.6 (5–100)	23.1 ± 13.0 (5–70)	35.1 ± 17.8 (11–100)	**<0.001**
Distant metastasis at diagnosis	4	1	3	NS*
pT stage				
T1/T2/T3	36/63/31	27/33/6	9/30/25	**<0.001**
pT1	36	27	9	**<0.001**
pT2	63	33	30	NS*
pT3	31	6	25	**<0.001**
pN stage				
NX/N0/N1	129/0/1	66/0/0	63/0/1	NS*
pM stage				
M0/M1	126/4	65/1	61/3	NS*
Multifocal disease	4	2	2	NS*
Microcarcinoma	7	5	2	NS*
HCC	63	25	38	**<0.05**
Concomitant PTC	21	15	6	NS*
Follow-up yes/no	117/13	62/4	55/9	NS*
Follow-up (±SD^a^) (range) months	103.9 ± 39.5 (17–182)	95.5 ± 111.0 (17–174)	104.5 ± 109.5 (27–182)	NS*
RAI/no RAI	105/12	54/8	51/3	NS*
Distant metastasis at follow-up	3	0	3	NS*

SD^a^: standard deviation; NS*: not significant.

**Table 2:** HCC versus non-HCC patients: clinical, pathological, and follow-up characteristics.

	HCC	Non-HCC	*P* value
Patients	63	67	
Age (±SD^a^) (range) years	55.0 ± 14.4 (18–85)	47.5 ± 13.8 (21–88)	<0.01
Male/female	18/45	21/46	NS*
Tumour size (±SD^a^) (range) mm	32.4 ± 18.9 (6–100)	25.6 ± 13.5 (5–65)	<0.05
Distant metastasis at diagnosis	1	3	NS*
pT stage			
T1/T2/T3	14/30/19	22/33/12	NS*
pN stage			
NX/N0/N1	62/0/1	67/0/0	NS*
pM stage			
M0/M1	62/1	64/3	NS*
MI/WI	25/38	41/26	<0.05
Multifocal disease	3	1	NS*
Microcarcinoma	4	3	NS*
Concomitant PTC	8	13	NS*
Follow-up yes/no	58/5	59/8	NS*
Follow-up (±SD^a^) (range) months	101.9 ± 44.6 (17–179)	104.8 ± 34.8 (29–174)	NS*
RAI/no RAI	55/3	50/9	NS*
Distant metastasis at follow-up	3	0	NS*

SD^a^: standard deviation; NS*: not significant.

**Table 3:** M1 versus M0 patients: clinical, pathological, and follow-up characteristics.

	M1	M0	*P* value
Patients	6	124	
Age (±SD^a^) (range) years	59.8 ± 16.2 (36–80)	50.7 ± 14.4 (18–88)	NS*
Male/female	4/2	35/89	NS*
Tumour size (±SD^a^) (range) mm	33.3 ± 15.6 (20–50)	28.7 ± 16.7 (5–100)	NS*
pT stage			
T1/T2/T3	1/1/4	22/24/15	NS*
pN stage			
NX/N0/N1	6/0/0	123/0/1	NS*
MI/WI	1/5	66/58	NS*
HCC	3	60	NS*
Multifocal disease	0	4	NS*
Microcarcinoma	0	7	NS*
Concomitant PTC	1	20	NS*
Follow-up yes/no	5/1	112/12	NS*
Follow-up (±SD^a^) (range) months	106.2 ± 49.6 (57–173)	104.7 ± 39.2 (17–182)	NS*

SD^a^: standard deviation; NS*: not significant.
